# Real-time monitoring by interferometric light microscopy of phage suspensions for personalised phage therapy

**DOI:** 10.1038/s41598-024-79478-w

**Published:** 2024-12-30

**Authors:** Benjamine Lapras, Camille Merienne, Emma Eynaud, Léa Usseglio, Chloé Marchand, Mathieu Médina, Camille Kolenda, Thomas Briot, Frédéric Laurent, Fabrice Pirot, PHAGEinLYON, Benjamine Lapras, Camille Merienne, Emma Eynaud, Léa Usseglio, Chloé Marchand, Mathieu Médina, Camille Kolenda, Thomas Briot, Frédéric Laurent, Fabrice Pirot

**Affiliations:** 1 Pharmacy Department, Hospices Civils de Lyon, Hôpital E. Herriot, Plateforme FRIPHARM, 69437 Lyon, France; 2 Laboratoire de Pharmacie Galénique Industrielle, Faculté de Pharmacie, Université Claude Bernard Lyon 1, 8, avenue Rockefeller, 69008 Lyon, France; 3 Tissue Biology and Therapeutic Engineering Laboratory (LBTI), CNRS UMR 5305, 69007 Lyon, France; 4 Bacteriology Department, Hospices Civils de Lyon, Hôpital Croix Rousse, French National Reference Centre for Staphylococci, 69317 Lyon, France; 5 Claude Bernard Lyon 1 University, Centre International de Recherche en Infectiologie (CIRI), INSERM U1111, CNRS UMR 5308, 69365 Lyon, France; 6 Pharmacy Department, Hospices Civils de Lyon, Hôpital Croix Rousse, 69317 Lyon, France; 7 Laboratoire d’Automatique, de Génie Des Procédés et de Génie Pharmaceutique, Université Claude Bernard Lyon 1, CNRS UMR5007, 69622 Villeurbanne, France

**Keywords:** Bacteriophages, Therapeutic phage suspension, Virus quantification, Fast purification monitoring, Viral stability, Aggregation, Antimicrobials, Characterization and analytical techniques

## Abstract

Phage therapy uses viruses (phages) against antibiotic resistance. Tailoring treatments to specific patient strains requires stocks of various highly concentrated purified phages. It, therefore, faces challenges: titration duration and specificity to a phage/bacteria couple; purification affecting stability; and highly concentrated suspensions tending to aggregate. To address these challenges, interferometric light microscopy (ILM), characterising particles (size, concentration, and visual homogeneity) within minutes, was applied herein to anti-*Staphylococcus aureus* myovirus phage suspensions. Particle concentration was linearly correlated with phage infectious titre (R^2^ > 0.97, slope: 3 particles/plaque forming units (PFU)) at various degrees of purification, allowing to approximate the infectious titre for suspensions ≥ 3 × 10^8^ PFU/mL, thereby encompassing most therapeutic doses. Purification narrowed and homogenised particle distribution while maintaining therapeutic concentrations. When compared to dynamic light scattering, electrophoretic mobility, and UV/Visible-spectroscopy, ILM best detected aggregates according to our homemade scoring. Although ILM has certain limitations, such as the inability to detect podoviruses (hydrodynamic diameter < 80 nm), or to measure particles in low-concentrated suspensions (< 10^8^ particles/mL), the present proof-of-concept positions this technique as a valuable quality control tool, as a complement to titration rather than a replacement for this technique, for phage suspensions, paving the way for further investigations.

**Supplementary Information:**

The online version contains supplementary material available at 10.1038/s41598-024-79478-w.

## Introduction

According to the World Health Organization, the projected death toll due to antibiotic resistance is expected to reach 10 million cases per year by 2050^1^. This alarming prediction drives the research for antimicrobial agents, of which phage therapy is amongst the most promising and clinical applications have been increasingly reported (clinical trials and case reports) since the beginning of the century^2^. Phage therapy uses natural viruses, known as tailed bacteriophages or phages, of approximately 50 to 200 nm in size, that have the ability to selectively infect, and rapidly kill bacteria. Each phage strain has a narrow spectrum compared to antibiotics, allowing for targeted treatment that minimises microbiome disruption and limits selection pressure^3^. Consequently, one approach to phage therapy is to select phages for their activity against a specific patient’s bacterial strain in order to provide a tailored drug product^4^.

This personalised strategy requires access to stocks of various highly concentrated and purified phages amongst which can be selected those specific to a patient’s strain. Maintaining such stocks can be challenging. Phages are typically produced by infecting a bacterial strain, inducing bacterial lysis and releasing progeny. This process requires the elimination of bacterial impurities (e.g., toxins, metabolites and cell wall debris) with a subsequent purification stage that has to achieve sufficient phage yield in order to preserve therapeutic concentrations (commonly between 10^7^ and 10^9^ PFU/mL)^5–8^. However, the concentration of active phages can be impaired during purification and storage by changes in pH, salt concentration^9^, mechanical stress^10^, or simply steric hindrance associated with high concentrations^11^. These phenomena induce a shift of the thermodynamic equilibrium through the loss of the phage genetic material induced by an osmotic stress^12^, or the modification of surface charges, thereby altering the native structure of phage coat proteins to create new interactions, leading to phage aggregation, denaturation, and adsorption to surfaces^13,14^. This jeopardises the quality of the final pharmaceutical product. Therefore, either on-line or fast off-line monitoring of phage suspensions is required to avoid loss of time and undue costs.

**Fig. 1:**
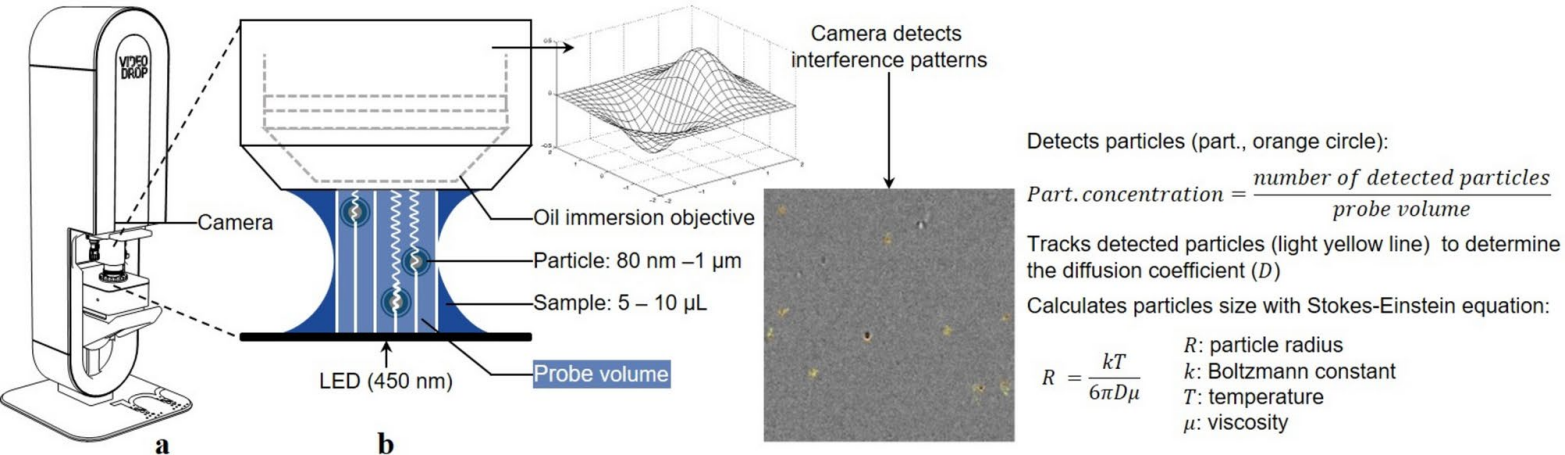
Principle of interferometric light microscopy (ILM) measurement. (**a**) and (**b**) adapted from Myriade communications.

The concentration of active phages (phage titre or infectious titre) is determined by a biological titration method. For this, a specific phage/bacteria couple is incubated for approximately 18 h^7,15,16^. This technique presents two major issues when applied to maintaining stocks of various highly concentrated and purified phages for personalised therapy: (i) the duration renders titration unsuitable for the swift monitoring of phage suspensions during purification; (ii) the specificity of the phage/bacteria couple implies an adaptation of the technique to each phage. However, recently developed technologies—or adapted from other fields–could address these issues by enabling real-time (in a few minutes) quantification of viral particles^17^ without the need to adapt the technique to a specific virus. These technologies often provide additional information such as particle diameter^17^. The following methods have been reported to characterise viruses^17–28^, extracellular vesicles^21^, and protein aggregates^29–31^: spectroscopic methods (e.g., turbidity^29,30^; UV/Visible-spectroscopy^29–32^); scattering methods (e.g., small-angle X-ray scattering, SAXS^29^; multi-angle laser light scattering, MALLS^17,29^; static light scattering, SLS^31^); combined light scattering and Brownian motion methods (dynamic light scattering, DLS^19,20,29^; nanoparticle tracking analysis, NTA^17,18,21,29^; interferometric light microscopy, ILM^21,22,28^); optical, epifluorescence, electronic, and atomic force microscopy^23–26,29,33^; flow cytometry (FCM)^17–19,27^; and quantitative real-time polymerase chain reaction (qPCR)^18,19,29^. When applied to phage suspensions, such characterisation (suspended particle concentration and size distribution) could monitor the purification performance and help understand the degradation pathways, such as aggregation, during storage. In this regard, the method selected for phage characterisation should address the following specifications: (i) swift (maximum a few minutes) assessment of viral particles (ii) size, (iii) count and (iv) detection of aggregates. These specifications are met only by DLS, NTA and ILM (Table 1). Recently, the rapid assessment of purified phage suspension for stability testing and titre loss prediction using DLS has been reported^20^, as has NTA and ILM for the quantification of purified phage particle concentration and size distribution^21^. However, DLS and NTA are unsuitable to analyse highly polydispersed samples^19^ (i.e., unpurified samples), whereas ILM has successfully been applied to complex samples such as river water^28^ and various stages of lentivirus and adenovirus preparation^22^. When illuminated from below, particles in suspension interact with the incident light, generating interference patterns that ILM can detect, count and track over a few seconds. For particles with sizes in the range of 80 nm to 1 µm, ILM allows the determination of the particle concentration (above 10^8^ particles/mL), and their size distribution, while providing the imaging of the interference pattern (Fig. 1). To our knowledge, there are no reports of ILM used to approximate the infectious phage titre, monitor phage purification or detect phage aggregates.

**Table 1:** Methods typically used to characterise particles (viruses, extracellular vesicles, and protein aggregates) and their ability for phage counting, size distribution characterisation, and detection of aggregates in suspension.

Methods	Measurements			Analysis time
	Counting	Size distribution	Aggregation	
Spectroscopy^29–31^				
Turbidimetry	No	No	Yes	< 10 min
UV/Vis-spectroscopy	No	No	Yes	< 10 min
Scattering^17,29,31^				
SAXS, MALLS, SLS	No	Yes	Yes	Min to hour
Combined light scattering and Brownian motion^17–22,28,29,31^				
DLS	Yes	Yes	Yes	15–20 min
NTA	Yes	Yes	Yes	15–20 min
ILM	Yes	Yes	Yes	< 10 min
Microscopy^17–19,23–26,29,33^				
Optical	No	No	Yes	15–20 min
Epifluorescence	Yes	No	Yes	Hours
Electronic	No	Yes	Yes	Hours
AFM	No	Yes	Yes	Hours
Miscellaneous^17–19,27,29^				
FCM	Yes	Yes	Yes	Min to hour
qPCR	Yes	No	No	Hours
Plaque assay	Yes	No	No	Hours

Small-angle X-ray, SAXS; Multi-angle laser light scattering, MALLS; Static light scattering, SLS; Dynamic light scattering, DLS; Nanoparticle tracking analysis, NTA; Interferometric light microscopy, ILM; Atomic force microscopy, AFM; Flow cytometry, FCM; Quantitative real-time polymerase chain reaction, qPCR.

In the present study, a proof-of-concept of the suitability of ILM for real-time monitoring during the production and storage of personalised phage medicine was performed. Myovirus phage suspensions, that are in the instrument detection size range, are analysed (particle concentration, size and ILM visual aspect) at various purification steps and results are compared with the plaque assay for quantification, and with DLS, electrophoretic mobility and UV/Vis-spectroscopy for aggregation detection. The innovative aspects covered in this paper, while applied to phage therapy, could be relevant to other viruses, nanoparticles, nanoemulsions, or quantum dots.

## Results

### Comparison of phage quantification by ILM and spot-test titre

Particles dispersed in dilutions (10 samples ranging from 3 × 10^8^ to 3 × 10^9^ particles/mL) of a purified phage suspension, were quantified using a spot-test (benchmark method; infectious titre, *x*_*i*_) and ILM (particle concentration, *y*_*i*_). Results were compared according to the methodology of the NF EN ISO 15,189:2022 standard.

**Fig. 2:**
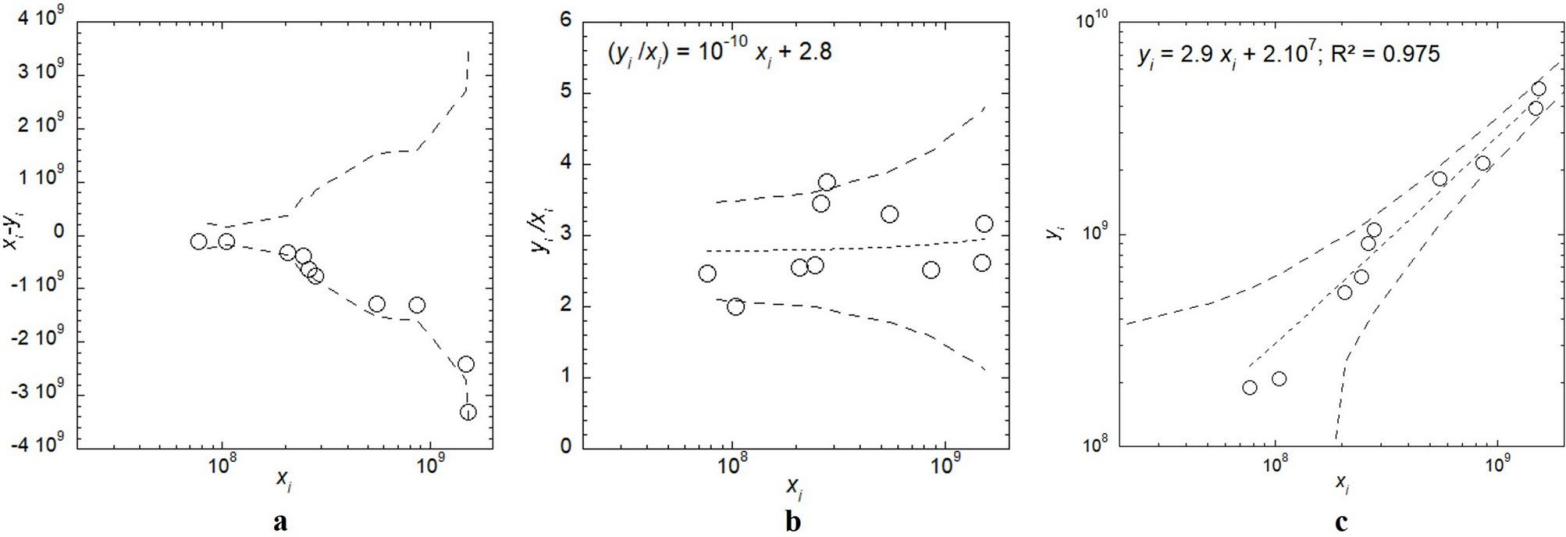
Comparison of (*x*_*i*_) phage infectious titre (PFU/mL) and (*y*_*i*_) particle concentration (particles/mL) (N = 10 experimental data; o). (**a**) Difference plot. Dashed lines represent the acceptable limits, i.e., $Limits=\pm \sqrt{{\left(3{\sigma }_{{y}_{i}}\right)}^{2}+{\left(3{\sigma }_{{x}_{i}}\right)}^{2}}$ according to standard NF EN ISO 15,189:2022 methodology. (**b**) Particle concentration and phage infectious titre ratio. The dotted line represents the linear regression fitted to the experimental data set and the dashed lines represent the limits of the 95% confidence interval. (**c**) Particle concentration as a function of phage infectious titre. The dotted line represents the linear regression fitted to the experimental data set and the dashed lines represent the limits of the 95% confidence interval. Statistical analyses: Levene’s test (*p* = 0.10), paired t-test (t_0.05,9_ = 2.26), hypothesis test for the slope (t_β1_ = 17.66), and hypothesis test for the intercept (t_β0_ = 0.15).

For each sample, the difference between phage infectious titre and particle concentration (*x*_*i*_*-y*_*i*_) indicated that all data were within acceptable limits and that they were negative (Fig. 2a). Therefore, the infectious titre (*x*_*i*_) was systematically lower than the particle concentration (*y*_*i*_).

A linear regression was performed on the ratio of each sample (*y*_*i*_*/x*_*i*_). It indicated that the particle concentration-to-phage infectious titre (*y*_*i*_*/x*_*i*_*)* ratio could be approximated to the constant value of ~ 3 particles/PFU. Of note, two out of the ten samples, with phage concentrations < 3 × 10^8^ PFU/mL, had a *y*_*i*_*/x*_*i*_ ratio outside the 95% confidence interval (Fig. 2b).

The correlation between the infectious titre (*x*_*i*_) and particle concentration (*y*_*i*_) indicated a linear relationship (*R*^2^ = 0.975, t_β1_ > t_0.05,9_), represented as *y*_*i*_ = ~ 2.9 *x*_*i*_, with each data falling within the 95% confidence interval (Fig. 2c).

Because of the demonstrated linearity between the measured particle concentration (*y*_*i*_) and phage infectious titre (*x*_*i*_), ILM could be employed to approximate the infectious titre by applying a correlation model (*x*_*i*_ = *2.9/y*_*i*_). With 6 values overestimated by the model and 4 values underestimated, the distribution of experimental data around this correlation model was considered homogeneous. The RMSE, or the error of the model (correlation error), was 8 × 10^7^ PFU/mL, corresponding to 2 × 10^8^ particles/mL. The correlation error is thus slightly higher than the lower detection limit communicated by the manufacturer of this instrument (1 × 10^8^ particles/mL).

The bias between ILM log_10_ (particle concentration) and log_10_ (phage infectious titre), ranged between 0.04 and 0.07 (mean value ± standard deviation (sd): 0.05 ± 0.01). The repeatability was 0.02 ± 0.01 for the infectious titre and 0.01 ± < 0.01 for particle concentration.

### In-process monitoring of phage purification by ILM

**Fig. 3:**
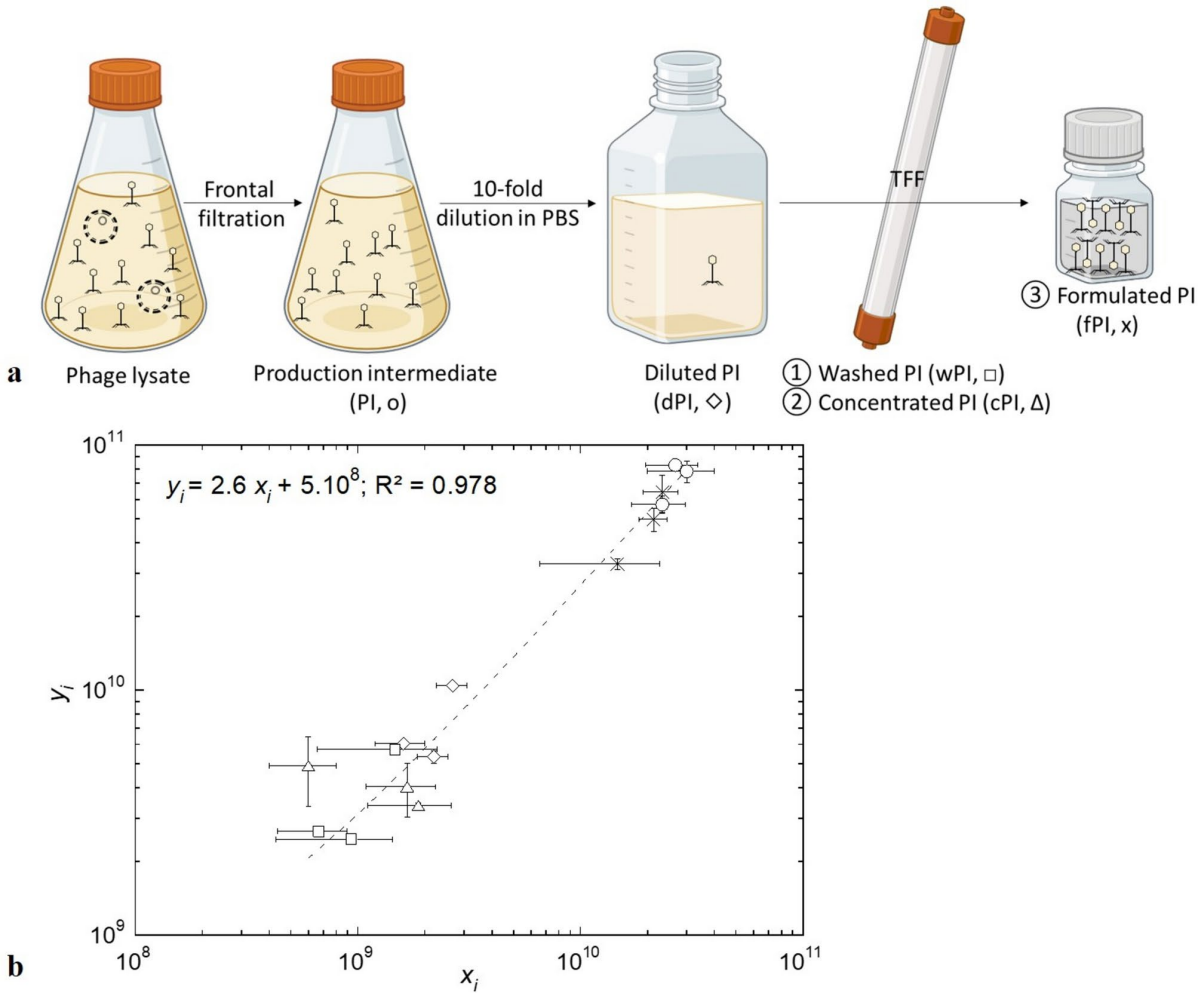
(**a**) Purification process flowchart (partly created with BioRender.com). The phage lysate undergoes a frontal filtration, forming a production intermediate (PI), which is then tenfold diluted in PBS, forming a diluted PI (dPI), which enters tangential flow filtration (TFF) where the phage suspension is washed (wPI), then 10-time concentrated (cPI) and finally formulated (fPI) in PBS. (**b**) Particle concentration (*y*_*i*_) as a function of phage infectious titre (*x*_*i*_) during three replicates of purifications: (o) production intermediate, PI; (◇) diluted PI, dPI; (□) washed PI, wPI; (Δ) concentrated PI, cPI; (x) formulated PI, fPI. Each point is the mean value of three experimental measurements ± standard deviation. The dashed line represents the linear regression fitted to the experimental data set (overall *N * = 15). Statistical analyses: Levene’s test (*p* = 0.08), paired t-test (t_0.05,14_ = 2.15), hypothesis test for the slope (t_β1_ = 23.74), and hypothesis test for the intercept (t_β0_ = 0.29).

A five-step purification process (frontal filtration, dilution, washing, concentration and formulation) was performed in triplicate (replicates A to C) on a phage lysate. At each step, samples (production intermediate, PI; diluted PI, dPI; washed PI, wPI; concentrated PI, cPI; formulated PI, fPI; Fig. 3a) were analysed in real-time by ILM and at the end of the process by spot-test titration.

A linear correlation (R^2^ = 0.978) between phage infectious titre (*x*_*i*_) and particle concentration (*y*_*i*_) was observed at various stages of the purification process (Fig. 3b), with a slope value (t_β1_ > t_0.05,9_) very close to that determined with data from purified suspensions (2.6 vs. 2.9 particles/PFU). Notably, a tenfold increase in both infectious titre and particle concentration of cPI compared to wPI would have been expected, which is not observed here. However, as these samples were collected during TFF without pausing the process, unlike other samples that are taken at the end of production steps (e.g., frontal filtration, dilution, TFF), the discrepancy can be attributed to differences in sampling techniques, especially since the particle concentration increases tenfold in the fPI sample, which represents the final TFF step.

**Fig. 4:**
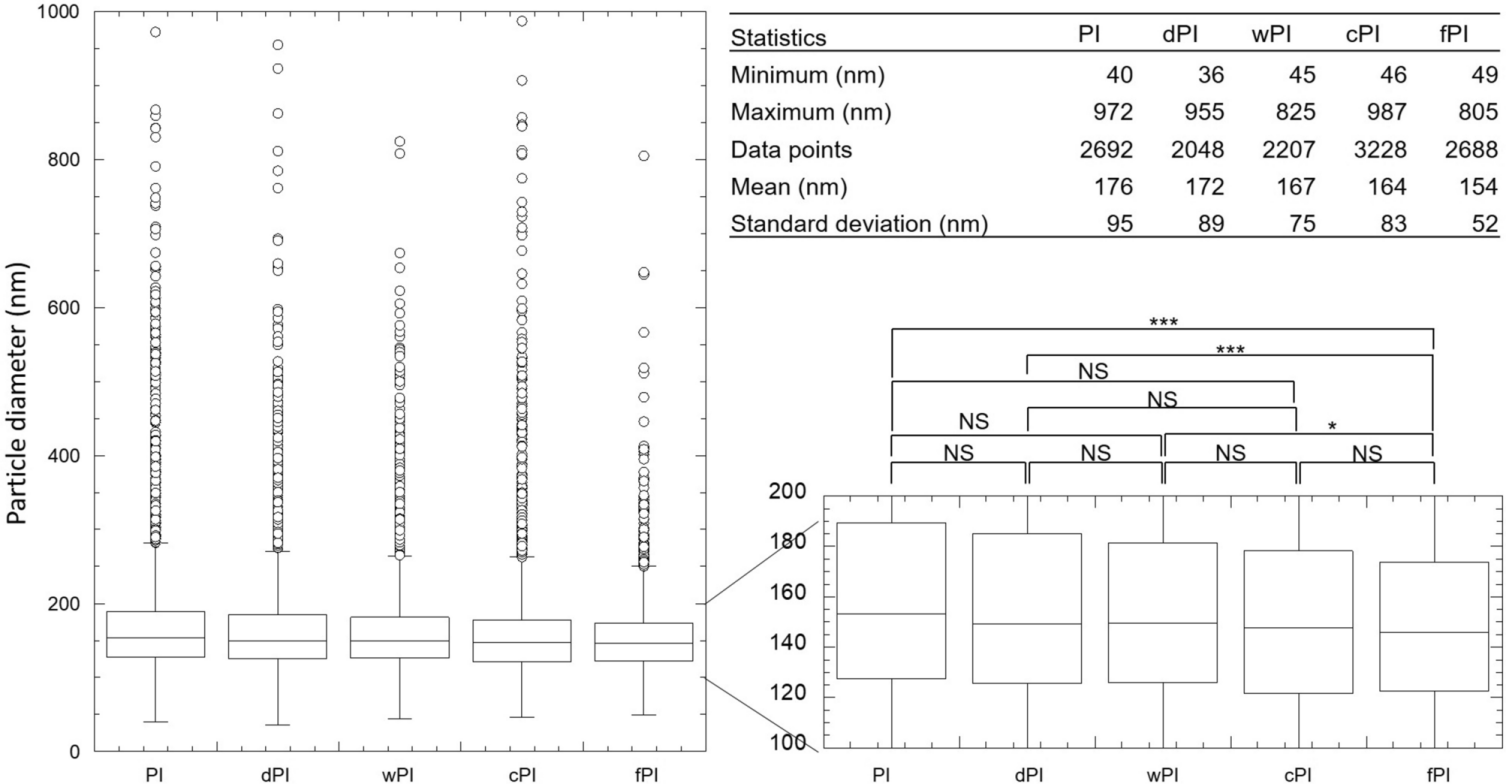
Size distribution of all detected particles by ILM for each purification step and all purifications (N = 3). The data set’s characteristics used for the box plot representation is depicted in the table in the top right corner. The line in the box plot represents the median. The homogeneity of variances was demonstrated with Levene’s test (*p* = 0.27). One way repeated measures ANOVA performed on all groups. All pairs comparison performed with Tukey HSD Post Hoc test: NS: not significant; * *p* < 0.05; ** *p* < 0.01; *** *p* < 0.001.

Although significant differences, in either phage infectious titre or particle count, were observed throughout purifications (all *p* < 0.001, Supplementary Table 1), the infectious titre and particle concentration at the beginning (PI) and the end (fPI) of the process were > 1 × 10^10^ PFU/mL or particles/mL for each purification replicate (Table 2 for replicate B; Supplementary Table 2 for replicate A; Supplementary Table 3 for replicate C). As an example, during the purification of replicate B, ILM monitoring showed the following general tendencies: a narrowing of the size distribution (visible on the diagram and through the decrease in the mean diameter of particles), due to the reduction in particles above 400 nm, and fewer aggregates and homogenisation of the suspension according to ILM imaging (Table 2). These tendencies were true for the other replicates as supported by the size distribution of all detected particles, showing a fPI box-plot distribution with fewer outliers’ points and a fPI mean diameter significantly different from the first three steps of the process (PI, dPI, wPI, *p* < 0.05). Additionally, the particle diameter (mean value ± sd) decreased from (176 ± 95) nm in the PI suspension to (154 ± 52) nm in the fPI (Fig. 4). Notably, the mean particle diameter of fPI differs from the TEM measurements (over 200 nm in length); however, this discrepancy can be attributed to differences in sample treatment, measurement methods, and the number of particles analysed per sample.

**Table 2:** In-process characterisation of purification replicate B by ILM and spot-test.

Particle characterisation	Purification steps				
	PI	dPI	wPI	cPI	fPI
ILM image					
Infectious titre^ǂ^ (PFU/mL)	(2.3 ± 0.6) 10^10^	(1.6 ± 0.4) 10^9^	(6.7 ± 2.3) 10^8^	(1.7 ± 0.6) 10^9^	(2.1 ± 0.3) 10^10^
Particle concentration^†^ (particles/mL)	(5.7 ± 0.5) 10^10^	(6.0 ± 0.1) 10^9^	(2.6 ± < 0.1) 10^9^	(4.0 ± 1.0) 10^9^	(5.0 ± 0.5) 10^10^
Mean particle diameter^†^ (nm)	(175 ± 8)	(171 ± 3)	(160 ± 8)	(166 ± 1)	(152 ± 3)
Size distribution diagram					

Each value is the (mean value ± sd) of 2 (†) to 3 (ǂ) experimental determinations. ILM image and size distribution diagram were taken on undiluted samples while particle concentration and mean size were measured on samples diluted to fit the instrument specifications. The particle concentration displayed here is the detected value multiplied by the sample’s dilution factor. On the ILM images, the orange lines circle detected particles (between 80 nm and 1 µm) and the yellow lines track the particle movement.

The lowest bias between log_10_ (particle concentration) and log_10_ (phage infectious titre) of 0.04 ± < 0.01 (mean ± sd) was associated with the fPI step. For PI, the bias was 0.04 ± 0.01. However, as the purification process progressed, the bias gradually expanded, reaching 0.06 ± 0.01 for the dPI and wPI steps, and ultimately 0.06 ± 0.04 for the cPI product.

In summary, particle counting by ILM, linearly correlated with infectious titre, can be continuously monitored throughout the purification process. This allowed the verification of particle concentration preservation at the end of the process. ILM also offered valuable insights into particle homogenisation, aggregate reduction, and size distribution refinement.

### Phage aggregate detection by ILM, DLS, electrophoretic mobility, and UV/Vis-spectral methods

**Fig. 5:**
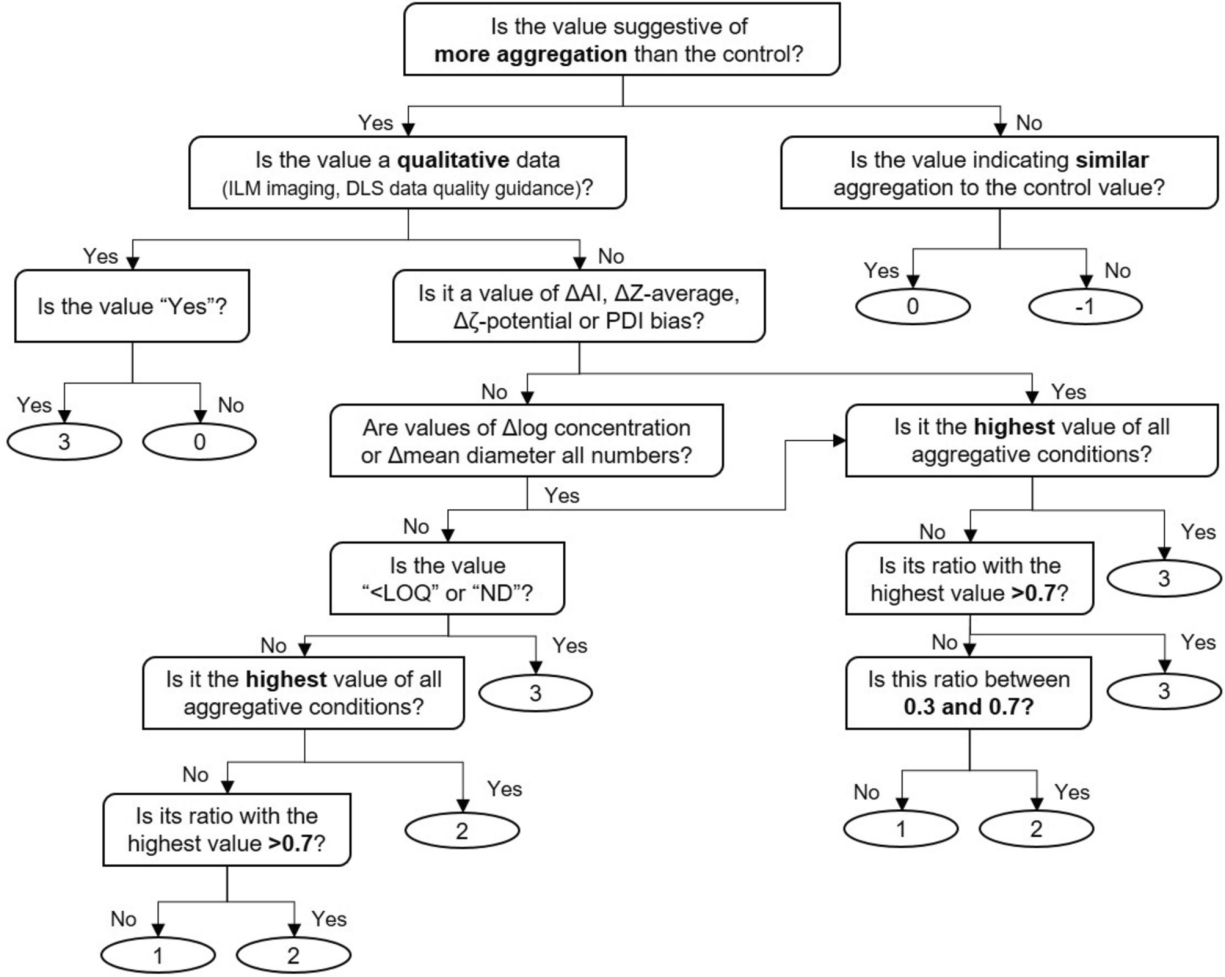
Algorithm for assigning a phage aggregation score (ranging from − 1 to 3) to the values obtained from the comparison with the control. Aggregation index, AI; polydispersity index, PDI; limit of quantification, LOQ; not detected, ND.

Various stresses (acidification, alkalinisation, 40 °C heating, and rotating agitation) were applied to phage suspensions to induce aggregation through different degradation pathways. The ability of ILM to detect the generated aggregates (via particle concentration; mean diameter; and imaging data) was compared to that of DLS (Z-average or the intensity-weighted harmonic mean hydrodynamic diameter; detection of “larger-sized populations”; polydispersity index, PDI), electrophoretic mobility (ζ-potential) and UV/Vis-spectral methods (aggregation index calculated with absorption at 255 and 350 nm) (Table 3). In this regard, an internal scoring system was elaborated. Briefly, scores ranging from − 1 (in favour of less aggregation than a control suspension kept refrigerated) to 3 (in favour of a strong aggregation compared to the control sample) were assigned to each measure (Fig. 5). Mean scores were then calculated for each method and each aggregative condition (Table 3).

**Table 3:** Characterisation of aggregation by four techniques.

Technique	pH		Agitation^*c*^	Heating (40° C)^*d*^	Technique mean score [^*a*^ to ^*d*^]
	3^*a*^	12^*b*^			
UV/Vis-spectral					1.3
ΔAI (%)	10 ± < 1	−5 ± 1	0 ± 2	8 ± 2	
Score #1	3	−1	0	3	
ILM					2.1
Δlog concentration (log(particles/mL))	< LOQ	−2 ± < 1	< LOQ	− 1 ± < 1	
Score #2	3	2	3	1	
ΔMean diameter (nm)	ND	−26 ± 4	ND	145 ± 13	
Score #3	3	−1	3	2	
Viewed particles > 1 µm	Yes	Yes	No	Yes	
Score #4	3	3	0	3	
DLS					1.8
ΔZ-average (nm)	4408 ± 566	101 ± 17	1256 ± 180	286 ± 59	
Score #5	3	1	2	1	
Larger-sized population	No	Yes	No	Yes	
Score #6	0	3	0	3	
Bias PDI	1.77 ± 0.37	2.27 ± 0.30	0.21 ± 0.16	0.48 ± 0.08	
Score #7	3	3	1	1	
Electrophoretic mobility					2.0
Δζ-potential (mV)	10	−7	10	9	
Score #8	3	−1	3	3	
Aggregative condition mean score [#1 to #8]	2.6	1.1	1.5	2.1	

Except for ζ-potential, which was only measured once, when quantitative, the mean value ± standard deviation of 3 experimental determinations of aggregative conditions (z) is compared to the mean value of 3 measures of the control condition (w): Δ = z-w; Δlog = log_10_(z)-log_10_(w); bias = (z-w)/w. When qualitative, a “Yes” or “No” value is attributed. A score is thereafter attributed from − 1 (in favour of less aggregation compared to the control) to 3 (strongly in favour of aggregation), with 0 being no difference from the control. “<LOQ” is attributed to the mean diameter value if the particle count is lower than the limit of quantification.

In aggregative conditions, the calculations were performed by comparison with a control:

*a* and *b*: AI: 8%; 6 × 10^9^ particles/mL; particles mean size: 162 nm; Z-average: 131 nm; PDI: 0.18; ζ-potential: − 11 mV.

*c* and *d*: AI: 19%; 2 × 10^9^ particles/mL; particles mean size: 187 nm; Z-average: 175 nm; PDI: 0.37; ζ-potential: − 15 mV).

AI = Aggregation index; PDI = Polydispersity index; LOQ = Limit of quantification; ND = Not detected.

when "< LOQ” is attributed to Δlog concentration, the number of particles detected is too low to accurately estimate the particle size distribution (< 10^8^ particles/mL). Consequently, “ND” is assigned to the Δmean diameter.

The acidic condition demonstrated the highest level of detected aggregation (mean score: 2.6), followed by heating (mean score: 2.1), whereas the lowest levels of detected aggregation were observed during agitation (mean score: 1.5) and alkalinisation (mean score: 1.1).

**Fig. 6:**
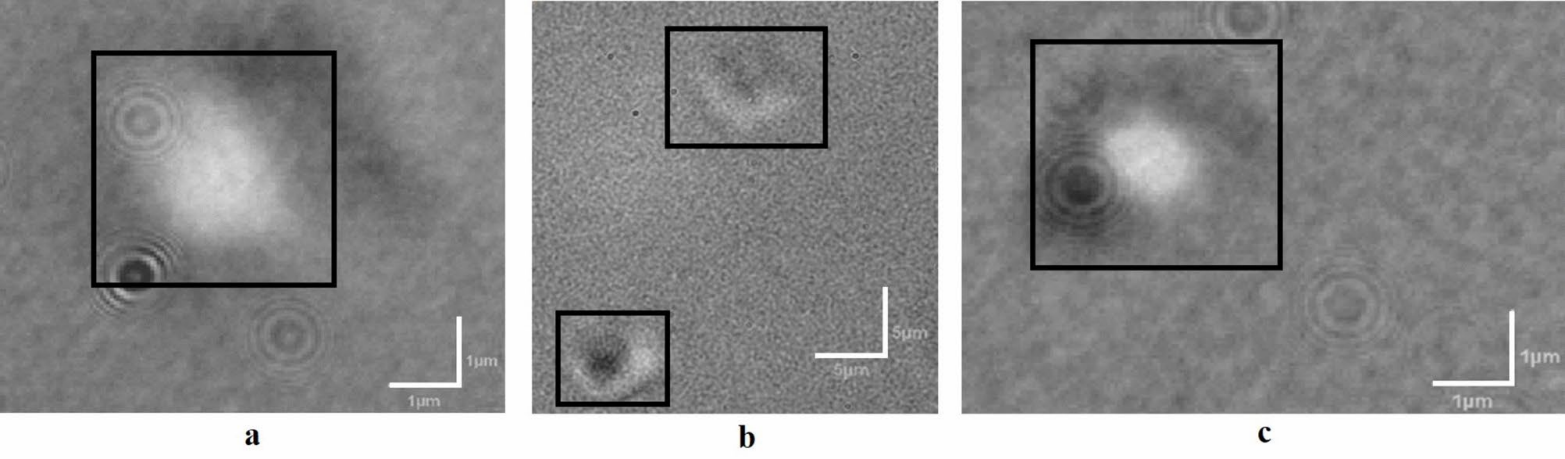
Screenshots of aggregates (outlined in black) viewed with ILM images for different aggregative conditions. The images are obtained by adjusting the viewing parameters of the software between the interference patterns (background formed by black and white dots) and the optical microscopy image (scale provided at the bottom right of the image and retraced to improve readability). (**a**) Suspension acidified to pH 3 (mean aggregative score of 2.6/3.0); scale bars, 1 µm. (**b**) Suspension alkalised to pH 12 (mean aggregative score of 1.1/3.0); scale bars, 5 µm. (**c**) Suspensions maintained for 4 days at 40 °C (mean aggregative score of 2.1/3.0); scale bars, 1 µm. Some artefacts are visible in the images, particularly in images (**a**) and (**c**), i.e., concentric circles.

ILM, directly imaging aggregates (Fig. 6), was rated with the highest score for detecting aggregates (mean score: 2.1), closely followed by electrophoretic mobility (mean score: 2.0) and DLS methods (mean score: 1.8). The UV/Vis-spectral method was the least efficient for detecting aggregates (mean score: 1.3).

## Discussion

The comparison of ILM particle concentration to spot-test infectious titre revealed that within the Videodrop® detection range (from 1 × 10^8^ to 1 × 10^10^ particles/mL), ILM assessed phage infectious titre with a consistent bias of 5% of log_10_-transformed values and, a good correlation (R^2^ > 0.97) with a particle-to-phage ratio of 3 for purified and unpurified myovirus phage suspensions. This indicates that the infectious activity of phages can be assimilated to a colligative property of phage suspensions. The phage-particle ratio might be explained by (i) the persistence of non-infectious colloidal particles in the purified sample (e.g., empty phage capsids or altered phage fibres), (ii) the inability of titration to detect all active particles (e.g., phages reversibly aggregated), or (iii) that a single plaque may be induced by several virions (i.e., virion clumping or secondary infection^33^). The latter would indicate that only a third of phages were capable of forming plaques, which is equivalent to an absolute efficiency of plating (EOP) of a third^33^. The EOP is influenced by adsorption rate and burst size, rendering it dependent on phage-bacteria specific characteristics. Hence, additional investigations are warranted on other phage-bacteria couples to assess the robustness of ILM as a rapid and dependable method for phage counting within the context of quality control procedures. Further studies should specifically investigate the interference of LPS micelles, which are similar in size to phages, in the analysis of production intermediates of phages targeting Gram-negative bacteria.

Interestingly, for several virus-derived vectors, the European Pharmacopoeia recommends determining their particle concentration as well as their infectious titre during final production stages (production harvest and purified harvest) in the general chapter *Gene transfer medicinal products for human use (5.14)* (04/2019:51,400). If such a recommendation is not explicitly made in the general chapter *Phage therapy active substances and medicinal products for human and veterinary use (5.31),* ILM could be useful, conjointly to phage titration. This approach might help prevent the administration of numerous non-infectious phages, which could otherwise contribute to an anti-phage immune response.

The observed linear correlation between phage infectious titre and particle concentration enables the development of a model, approximating the infectious titre of a suspension based on its particle concentration measured with ILM. Considering (i) previously reported constraints of Videodrop®^21^, i.e., its inability to detect phages of small sizes (e.g., podoviruses < 80 nm) or particle concentrations below its lower detection limit of 1 × 10^8^ particles/mL, (ii) the calculated RMSE (i.e., correlation error) of 8 × 10^7^ PFU/mL (equivalent to 2 × 10^8^ particles/mL), and (iii) that two points were out of the 95% confidence interval for concentrations < 3 × 10^8^ PFU/mL in the difference plot (Fig. 2b), the limit of the correlation model should be set ≥ 3 × 10^8^ PFU/mL. Consequently, for treatment protocols using phage doses < 1 × 10^8^ PFU/mL^8^, the assessment of phage infectious titre with ILM is unsuitable. However, it is generally accepted that the dose treatment of phages should be at least of 1 × 10^8 ^PFU/mL^7^ and therefore within the range of the model. Keeping these limitations in mind moving forward, the direct analysis of phage suspensions, without the necessity of background subtraction or the need of standard curves, proved to be straightforward, particularly when dealing with a minimal number of samples. Consequently, this method exhibits significant potential in overcoming the current challenge of titration duration, by providing, in a matter of minutes, the particle concentration in a phage suspension, that can be used to approximate the infectious titre. This capability is particularly promising for counting phages (other than podoviruses) in highly concentrated purified phage suspensions compounded immediately prior to patient administration.

ILM can be applied to any liquid sample, regardless of its viscosity (provided it is known, in order to correctly apply Stokes–Einstein’s equation) or purity and requires samples of only a few microlitres necessitating minimal preparation (dilution). Additionally, the data rapidly generated by ILM (i.e., imaging, particle concentration and size distribution) serve as valuable indicators for estimating the purification performance. In instances where (i) fewer aggregates are observed, with particles appearing homogeneously distributed in ILM images, (ii) the size distribution narrows due to a reduction in large-sized particles as indicated by the reduction of the mean diameter of particles, and (iii) the number of particles is preserved at the end of the process, then, collected data supports the purification performance. ILM is therefore particularly noteworthy for monitoring the purification process. This could reduce the risk of further processing an unsuccessful purification–e.g., low infectious titre, insufficient bacterial impurities reduction–resulting in limitation of financial losses. Indeed, purification affects the thermodynamic state of phage suspensions, making them prone to destabilisation, and therefore to activity loss. Hence, the correlation between the particle concentration measured with ILM and the infectious titre, its ability to assess the purification performance by a swift measure, positions ILM as a safety net during the downstream process. Of note, in this context, the assessment of purification success relies exclusively on particle concentration and size. If the process results in phage inactivation while preserving its structure (e.g., emptying the capsid of its genome), it may not be detected by ILM. Further experiments should be conducted to explore the capability of ILM to identify inactive phages with preserved mean size and to differentiate phages from protein aggregates. In this regard, as a preliminary step, subsequent studies could investigate the ability of ILM to distinguish ghost phages (with empty capsids) induced by an osmotic shock^34^ or phages inactivated by other means (e.g., high temperatures, typically above 50 °C, to induce genome ejection from the capsid; UVC radiations to damage nucleic acids by forming free radicals)^12^ from a negative control of purified phage suspensions. This differentiation could be achieved by analysing the light intensity detected by ILM for each particle, since ghost phages are expected to interfere with the incident light differently than full phages.

Maintaining the thermodynamic state of proteins is a challenge during the storage of highly concentrated and purified phage suspensions, which tend to undergo aggregation. Although size exclusion chromatography is used as a standard method for aggregate and fragment analysis of monoclonal antibodies, its resolution is too low to discriminate small aggregates from single particles for large molecules such as phages (e.g., the relative molecular mass of T4-like phages is ~ 210 M^35^). Therefore, in the present study, other strategies to detect aggregates (ILM, DLS, electrophoretic mobility and UV/Vis-spectral methods) were investigated under various (mechanisms and generation rates) pro-aggregative conditions. Variations in pH result in modifications of surface charges thereby altering electrostatic interactions. The initial ζ-potential of the phage was − 13 mV, indicating a predominantly negative charge. Acidification to pH 3 increased the ζ-potential to −1 mV, suggesting that phages were close to their isoelectric point. At this point, the surface charges of the coat proteins of phages neutralise, reducing repulsive electrostatic interactions that are overbalanced by attractive forces causing fast phage flocculation^36^ followed by strong aggregation, as indicated by the highest aggregation score associated with the acidic condition (mean score of 2.6/3.0). This aggregation score derives from a high aggregative index (AI), a reduction in particle concentration associated with the observation of particles larger than 1 µm with ILM, and a high Z-average and polydispersity index (PDI). Considering the lack of detection of larger-sized populations on DLS, these observations are all consistent with the flocculation of particles at low pH, forming agglomerates, whose size likely falls below the upper detection limit of the DLS instrument. Alkalisation to pH 12 decreased the ζ-potential to −18 mV indicating less positive surface charges. In this case, phage proteins may unfold before forming new structures with better thermodynamics, such as aggregates. With a lower AI and mean diameter due to chemical denaturation, but a higher PDI and aggregates observed on ILM imaging suggesting aggregation, it can be inferred that the alkaline conditions encompassed a mixed population of denatured and aggregated forms, which could explain the full spectrum of observed scores (from −1 to 3). This explanation is consistent with the low aggregation score associated with alkalinisation (mean score of 1.1/3.0). Another stress, 40 °C heating, induces an increase in kinetic energy, increasing Brownian motion and thereby, collisions between particles, making phages more prone to aggregate^36^ without inducing thermal denaturation, expulsion of the genome for the capsid observed at high temperatures^12^, or other modifications of the phage morphology. This is indicated by the high AI score, the elevated mean diameter and Z-average value, and the observation of particles larger than 1 µm with ILM and larger-sized population detected by DLS. However, the fact that the particle concentration is reduced but still detectable, and that the PDI is only slightly elevated, suggest that, at this temperature, the aggregation is more moderate than for the acidification condition, resulting in a mean score of 2.1/3.0. Rotating agitation induces aggregation by projecting particles onto the vial surface, causing denaturation and surface adsorption, followed by the ripping off of phage from the surface^37^. After 2 days of agitation, the data reported herein indicates a significant reduction in particle concentration, which is not correlated with an increase in AI or the observation of aggregates on ILM images. This suggests that most of the single particles and aggregates were adsorbed onto the packaging surface. However, with a significant increase of Z-average, a slight increase in PDI and a great increase of the ζ-potential (in favour of aggregation), it is likely that no aggregates either entered the focus of the microscope, or were present in the 7 µL samples, or that the aggregates were reversible and difficult to observe with ILM, explaining the aggregation score associated with agitation (mean score of 1.5/3.0).

According to our internal scoring, ILM (mean score of 2.1/3.0) was the best method for detecting aggregates closely followed by electrophoretic mobility (2.0/3.0) and DLS (1.8/3.0). Amongst the methods tested, UV/Vis-spectrophotometry was the least performant in detecting aggregates (1.3/3.0). This was mostly due to the high score attributed to direct imaging of aggregates and significant particle concentration reduction. However, for one of the aggregative conditions tested (2 days of rotating agitation), no aggregates were viewed on the ILM image whereas aggregates larger than 1 µm were detected by DLS (ΔZ-average of 1256 nm), suggesting that ILM’s ability to detect aggregates may depend on the degradation pathway. This proof-of-concept indicates the potential use of ILM to detect the formation of aggregates in forced degradation conditions. Further investigation should be carried out to increment its potential use for shelf-life stability testing, e.g., by assessing whether the correlation between the infectious titre and particle concentration is preserved through time, as phages slowly inactivate.

Interestingly, the ability of ILM to measure particle concentration and size in viscous and unpurified samples could be leveraged to detect the emergence of phages propagated on a bacterial strain in a liquid medium. Therefore, further investigations should be conducted to extend the application range of this technique to phage susceptibility testing (detection of active phages against a patient’s pathogenic bacterial strain for personalised therapy^7^), phage discovery (by selecting environmental samples containing active phages) and stability testing. While ILM is not suitable for detecting small phages and analysing suspensions < 10^8^ particles/mL, its ability to characterise highly concentrated anti-Gram positive bacteria phage suspensions, whether purified or unpurified, positions it as a promising quality control tool essential for the rapid development of personalised phage therapy.

If the biological titration method is the critical quality attribute of phage suspensions and remains mandatory, its duration is a hindrance to the use and expansion of phage therapy. The rapid (in a few minutes) approximation of phage infectious titre through the correlation with the particle concentration, monitoring of purification and detection of induced aggregates by ILM was demonstrated in the present study on myovirus anti-*Staphylococcus aureus* phages. Such innovative technologies, rapidly characterising phages through their colloidal properties, can alleviate the development of patient-tailored phage drug products at multiple stages (e.g., purification monitoring, stability testing) in a cost- and time-efficient manner. Such innovations will accelerate the provision of personalised phage therapy and contribute to the ongoing battle against antibiotic resistance.

## Methods

### Bacterial host

P2SA225 *S. aureus* strain was obtained from the French National Reference Centre for Staphylococci (Hospices Civils de Lyon, Lyon, France) as previously described^38^.

### Phage production

Phages (vB_SauM-V1SA19 and vB_SauM-V1SA20 deposited in GenBank with accession codes ON814134 and ON814135 respectively; dsDNA viruses; family *Herelleviridae*, genus *Silviavirus*; morphotype myovirus; genome lengths of 138,507 and 136,919 bp respectively) were isolated from wastewater samples taken in Lyon (France)^38^. According to previous transmission electron microscopy (TEM) analysis, capsid size was from 60 to 90 nm, and the overall length was from 200 to 300 nm^38^ (Supplementary Fig. S1). They were incubated with the P2SA225 strain at a multiplicity of infection (MOI) of 10^–2^ in 1 L of Superior Broth™ (Athenaes, Baltimore, MD, US) in a 2.5 L Fernbach culture flask with sided baffles (Avantor™ VWR™, Radnor, PA, US) for 4 h at 37 °C and 180 rotations per minute (RPM) shaking^38^. The product collected at the end of the incubation is named a phage lysate.

### Phage purification

The obtained phage lysate underwent the following purification process: frontal filtration 0.8 µm / 0.2 µm on polypropylene capsule filter Polycap TC150 (Cytiva, Little Chalfont, UK), followed by tenfold dilution in phosphate-buffered saline (PBS; pH 7.4 Gibco™, Life Technologies LTD, Paisley, UK), and then tangential flow filtration (TFF) that allowed the washing, ten-time concentration and formulation in PBS of the phage suspension. At each step, 50 µL of the suspension was sampled and respectively designated as: production intermediate, PI; diluted PI, dPI; washed PI, wPI; concentrated PI, cPI; formulated PI, fPI (Fig. 3a). The fPI was stored in a 125 mL polyethylene terephthalate (PET) PharmaTainer™ container (Cellon SA, Bascharage, Luxembourg) at 2–8 °C. Note that the wPI and cPI samples were collected during TFF by opening a pore of the column. Since TFF is a continuous process, it was not paused to allow sample collection at the end of the step. Therefore, these samples may not be as representative of the step investigated in terms of particle concentration or phage titre as those collected at operations’ endpoints.

### Particle size and concentration measurement using ILM

The Videodrop® (Myriade, Paris, France) uses a LED positioned beneath a 5 to 10 µL sample to generate interference patterns through the interaction between suspended particles and incident light. A camera, coupled to an oil-immersion microscope objective, detects these patterns, quantifies them (allowing the particle concentration to be determined in the known probed volume), and tracks them by recording short videos to estimate their diffusion coefficient (allowing calculation of their hydrodynamic radius with Stokes–Einstein equation)^22^. The principle of this instrument is summarised in Fig. 1. The Videodrop® camera, saturated between 90 and 95%, detects particles from 80 nm to 1 µm when concentrated between 1 × 10^8^ and 1 × 10^10^ particles/mL. Additionally, particles with a diameter exceeding 1 µm and up to 10 µm can be visualised through ILM microscopic imaging.

Samples of 7 µL were analysed with the Videodrop® at room temperature. When the particle concentration was over 1 × 10^10^ particles/mL, samples were diluted in PBS to fit within the detection limits. Acquisitions were stopped by default after 300 particles were tracked or after 20 videos were recorded. When the particle concentration was low (close to 1 × 10^8^ particles/mL), the maximum number of videos was increased up to 40. When the particle concentration was high (close to 1 × 10^10^ particles/mL), the minimum number of tracked particles was increased to 1000.

### Particles size measurement with dynamic light scattering (DLS)

The Zetasizer® Ultra ZSU3305 equipped with DTS1070 cell (Malvern Instruments Ltd, Malvern, UK) detects nanometric, sub- and micronic particles with a multi-angle DLS method. The scattering, resulting from the interaction of the 633 nm wavelength incident laser beam with particles, is collected at 12° (forward scattering), 90° (side scattering) and 173° (back scattering).

Measurements were collected at a fixed position of 5.5 mm from the outer cell wall. They were analysed using ZS Xplorer software version 2.0.0.98 (Malvern Panalytical Ltd, Malvern, UK) and the physical and optical properties of water and protein as dispersant and dispersed material at 25 °C, respectively, to derive the size distribution results of the viral suspensions (i.e., Z-average or the “intensity-weighted harmonic mean hydrodynamic diameter”^39^; the polydispersity index, PDI; and the data quality guidance of measures–e.g., “larger-sized populations”). The instrument was equilibrated for 1 min before taking measurements. The signal of triplicate samples (0.7 mL each, at 25 °C) was automatically attenuated to prevent detector saturation but still exceeded 100k counts per second (cps), indicating that the samples were sufficiently concentrated for the instrument.

### Zeta (ζ-) potential measurement with electrophoretic mobility

The surface charge properties of phages were determined by the measurement of particle ζ-potential^40^, calculated from the electrophoretic mobility of particles (Zetasizer® Ultra ZSU3305 equipped with a 633 nm laser beam and DTS1070 cell) using the physical and optical properties of water and protein as dispersant and dispersed material at 25 °C, respectively. The instrument was equilibrated for 1 min. The signal of triplicate phage samples (0.7 mL each, at 25 °C) exceeded 10k cps, indicating sufficient sample concentration for analysis.

### Spot-test titration

Infective phage titre was determined using the spot-test titration method as follows^16^. A phage suspension sample was diluted eight times in tenfold increments in trypto-casein soy broth medium (bioMérieux, Marcy-L’Etoile, France). Drops of 5 µL of each dilution were then laid on a P2SA225 bacterial lawn and incubated overnight. The results were expressed in PFU/mL. Each dilution was incubated on bacterial lawns in triplicate.

### Ultraviolet (UV)/Visible-spectrophotometry for aggregation index (AI) determination

A UV/Vis-spectrum–ranging from 500 to 220 nm with a step of 1 nm and a speed scan of 200 nm/min–was acquired using a semi-micro UV-polymer cuvette (Brand®, Wertheim, Germany) filled with 1 mL of sample and a UV/Vis V-730/730BIO Jasco spectrophotometer (JASCO Corporation, Tokyo, Japan). PBS was used as blank and reference. The aggregation index (AI) was calculated according to Eq. (1) adapted from Gupta et Mahalakshmi, 2020, with the absorbance at 255 nm (${\lambda }_{255}$), replacing the original wavelength of 280 nm to correspond to the phage DNA/protein complex maximum of absorption, and at 350 nm (${\lambda }_{350}$) reflecting the suspension’s turbidity^32^.

1 $$AI=\frac{{\lambda }_{350}}{{\lambda }_{255}-{\lambda }_{350}}\times 100$$

### Comparison of phage quantification by ILM and spot-test titre

Samples were prepared from a single fPI suspension of vB_SauM-V1SA19 phage with an initial infectious titre of approximately 4 × 10^9^ PFU/mL. Its initial particle concentration was assessed by ILM. According to the result of this initial measurement, this suspension was serially diluted in PBS to reach the following theoretical concentrations (*N* = 5): 3 × 10^9^ particles/mL; 2 × 10^9^ particles/mL; 1 × 10^9^ particles/mL; 6 × 10^8^ particles/mL; 3 × 10^8^ particles/mL. Each sample was measured in triplicate using ILM and spot-test titration. This experiment was conducted by two different operators on two different days; overall N = 10 samples were analysed.

ILM performance to quantify the number of purified phages suspended in a sample was assessed by comparison with the measured phage infectious titre (described in “Spot-test titration” Section). ILM evaluated the concentration of particles suspended in a sample (*y*), while the biological spot-test titration method determined the infectious titre of a phage suspension (*x*). Techniques were compared according to the standard NF EN ISO 15,189:2022 methodology, using titration as the benchmark method. For each (*i*) sample, an absolute difference plot–Eq. (2)–was graphed with its associated limits calculated–Eq. (3)–by considering the standard deviation ($\sigma$). Then, the particle concentration-phage infectious titre ratio was plotted—Eq. (4). The degree of association between ILM and titration results was assessed through the coefficient of determination of the comparison plot, Eq. (5), through hypothesis tests for the slope and intercept (described in “Statistical analyses” Section), and by ensuring results were within the 95% confidence interval of the mean slope value. Particle concentration determined using ILM was compared to phage infectious titre through the bias calculation–Eq. (6). In addition, ILM repeatability was compared to that of titration using Eqs. (7.A) and (7.B).

2 $$f\left( {\overline{{x_{i} }} } \right) = \left( {\overline{{x_{i} }} - \overline{{y_{i} }} } \right)$$

3 $${\text{Limits}} = \pm \sqrt {\left( {3\sigma_{{y_{i} }} } \right)^{2} + \left( {3\sigma_{{x_{i} }} } \right)^{2} }$$

4 $$f\left( {\overline{{x_{i} }} } \right) = \overline{{y_{i} }} /\overline{{x_{i} }}$$

5 $$f\left( {\overline{{x_{i} }} } \right) = \overline{{y_{i} }}$$

6 $${\text{Bias}} = \frac{{\log_{10} \left( {\overline{y}} \right) - {\text{log}}_{10} \left( {\overline{x}} \right)}}{{\log_{10} \left( {\overline{x}} \right)}}$$

7.A $${\text{Repeatability}}_{x} = \frac{{\log_{10} \left( {\sigma_{xi} } \right)}}{{\log_{10} \left( {\overline{x}_{i} } \right)}}$$

7.B $${\text{Repeatability}}_{y} = \frac{{\log_{10} \left( {\sigma_{yi} } \right)}}{{\log_{10} \left( {\overline{y}_{i} } \right)}}$$

If the relation between $(x)$ and $(y)$ was linear, then Eq. (8) was used to approximate the infectious titre $(\widehat{x})$ from the particle count value. Then the obtained infectious titre value $(\widehat{x})$ was compared to the experimental titre $(x)$ for each $(i)$ point of the (*N* = 10) experimental data set using Eq. (9), i.e., the root mean square error (RMSE).

8 $$\widehat{{x_{i} }} = \frac{{y_{i} }}{slope}$$

9 $${\text{Root}}\;{\text{mean}}\;{\text{square }}\;{\text{error }}\left( {{\text{RMSE}}} \right) = \sqrt {\frac{{\mathop \sum \nolimits_{i}^{N} \left( {\widehat{{x_{i} }} - x_{i} } \right)^{2} }}{N}}$$

### In-process monitoring of phage purification by ILM

A single batch of vB_SauM-V1SA20 phage underwent purification (described in “Phage purification” Section) in triplicate (replicates A to C). At each purification step (PI, dPI, wPI, cPI, and fPI), duplicate samples were promptly analysed using ILM, while spot-testing was conducted at the end of the purification process in triplicates (described in “Spot-test titration” Section). To match the Videodrop® detection range, some samples required dilution (typically PI 50-fold, dPI tenfold, wPI undiluted, cPI undiluted, fPI 50-fold). The linear relationship between phage infectious titre (*x*) and particle concentration (*y*) was analysed through hypothesis tests for the slope and the intercept (described in the “Statistical analyses” Section). The bias between the mean particle quantification $(\overline{y })$ obtained with ILM and phage infectious titre $(\overline{x})$ was calculated at each stage using Eq. (6).

### Phage aggregate detection by ILM, DLS, electrophoretic mobility, and UV/Vis-spectral methods

As an initial assessment of the potential use of ILM in stability studies, its ability to detect aggregates generated under forced conditions was compared with that of DLS, electrophoretic mobility, and UV/Vis-spectral methods. A fPI suspension of vB_SauM-V1SA19 phage, initially maintained at 2–8 °C (i.e., “*w*”, control condition), was subjected to aggregative conditions “*z*” (i.e., extreme pH, agitation or moderate heating^11,41^) comprising the following scenarios: extemporaneously acidified to pH 3 or alkalinised to pH 12; subjected to rotating agitation at 40 RPM (equivalent to 0.7 Hz) for two days (Tube Rotator TR-200D, Antylia Scientific Ldt., Staffordshire, UK); stored for four days at 40 °C to induce aggregation without inducing thermal denaturation. Each suspension was housed in a 15 mL polystyrene conical container sealed with a polypropylene cap (Falcon™, Corning Incorporated–Life Science, Reynosa, Mexico). The detection of phage aggregates in chemically and physically stressed suspensions encompassed: AI determined by UV/Vis-spectral analysis, Eq. (1); particle concentration, mean size, and aggregates observed in the microscopic images by ILM; Z-average, PDI and quality guidance data determined by DLS; and ζ-potential determined by electrophoretic mobility.

The difference in particle concentration determined by ILM, in “*w”* (control) and “*z”* (aggregative conditions) groups was calculated using Eq. (10). For concentrations < 1 × 10^8^ particles/mL (i.e., lower than the limit of quantification, LOQ), a “ < LOQ” note was attributed.

10 $$\Delta log={\text{log}}_{10}(z)-{\text{log}}_{10}\left(w\right)$$

The difference of AI, mean diameter, Z-average and ζ-potential analysis determined by either spectral analysis or DLS, in *w*- and *z* groups was calculated using Eq. (11). For concentrations < LOQ, the mean diameter was quoted as not detected (“ND” note).

11 $$\Delta =z-w$$

The variation of PDI was calculated using Eq. (12):

12 $$Bias=\frac{{\text{log}}_{10}\left(\overline{z }\right)-{\text{log}}_{10}\left(\overline{w }\right)}{{\text{log}}_{10}\left(\overline{w }\right)}$$

Furthermore, for qualitative data recorded by ILM and DLS, a “Yes” or “No” quote was respectively attributed for the detection or non-detection of uncharacterised (out of the quantification range of the instrument) particles (i.e., particles larger than 1 µm; undetected “larger sized-population” as reported by the quality guidance of the DLS software in samples containing poorly-characterised larger material such as aggregates or dust).

Then, a score of aggregation—ranging from − 1 to + 3—was attributed to the previous calculations, such as: − 1 and 0 scores indicated lower and similar aggregation than the control, respectively; for the parameters “viewed particles > 1 µm”, “larger-sized population”, “Δlog concentration”, and “Δmean diameter” value score of 3 was allocated to qualitative notes in favour of aggregation (denoted as “Yes”, “ < LOQ”, or “ND”), whereas a score of 0 was assigned for the absence of aggregation (denoted as “No”); the highest scores of ΔAI, ΔZ-average, Δζ-potential, and PDI bias were assigned a value of 3. Then, to assign scores ranging from 1 to 3 to the remaining values, their ratios relative to the highest scores (previously assigned 3) were calculated. The scoring was as follows: 3 for a ratio > 0.7; 2 for a ratio between 0.3 and 0.7; 1 for a ratio < 0.3. To assign scores ranging from 1 to 2 to the lowest “Δlog concentration” and “Δmean diameter” values, their ratios in comparison to the highest scores (assigned as 2) were calculated as follows: 2 for a ratio > 0.7 and 1 for a ratio ≤ 0.7 (Fig. 5). Finally, the mean score for each aggregative condition and each method (ILM, DLS, electrophoretic mobility, and UV/Vis-spectrum) was calculated to facilitate the comparison of the techniques’ performance in detecting aggregates generated by various degradations.

### Statistical analyses

A threshold of 0.05 was selected for the *p*-value level of significance. For repeated measures (e.g., during in-process monitoring, serial dilutions), the homogeneity of variances was assessed with Levene’s test performed on medians. If variances were homogeneous, then either a one way repeated measures ANOVA was performed, or a paired t-test (for hypothesis tests for the slope and the intercept).

Statistical analyses were either performed with KaleidaGraph® version 3.6 (Synergy Software, Reading, PA, US), with Microsoft® Excel® 2016 version 16.0.5422.1000 (Microsoft, Redmond, WA, US) or manually. Specific tests used are detailed within the figure legends or table footnotes.

## Supplementary Information

Supplementary Information 1.

## Data Availability

Data is provided within the manuscript or supplementary information files. Otherwise, the data that support the findings of this study are available from the corresponding author upon reasonable request.

## References

[1] Interagency Coordination Group on Antimicrobial Resistance. No time to wait: securing the futur from drug-resistant infections. at https://www.who.int/antimicrobial-resistance/interagency-coordination-group/IACG_final_summary_EN.pdf (2019).

[2] Uyttebroek, S. *et al.* Safety and efficacy of phage therapy in difficult-to-treat infections: A systematic review. *The Lancet Infectious Diseases* **0**, (2022). 10.1016/S1473-3099(21)00612-5 35248167

[3] Principi, N., Silvestri, E. & Esposito, S. Advantages and limitations of bacteriophages for the treatment of bacterial infections. *Front. Pharm.* **10**, 457104 (2019). 10.3389/fphar.2019.00513 PMC6517696 31139086

[4] Verbeken, G. & Pirnay, J.-P. European regulatory aspects of phage therapy: Magistral phage preparations. *Curr. Opin. Virol.* **52**, 24–29 (2022). 34801778 10.1016/j.coviro.2021.11.005

[5] Ferry, T. et al. Phage therapy as adjuvant to conservative surgery and antibiotics to salvage patients with relapsing s aureus prosthetic knee infection. *Front. Med. (Lausanne)* **7**, 570572 (2020). 33304911 10.3389/fmed.2020.570572 PMC7701306

[6] Young, M. J. et al. Phage therapy for diabetic foot infection: A case series. *Clin. Ther.* **45**, 797–801 (2023). 37442654 10.1016/j.clinthera.2023.06.009

[7] Suh, G. A. et al. Considerations for the use of phage therapy in clinical practice. *Antimicrob. Agents Chemother.* **66**, e02071-e2121 (2022). 35041506 10.1128/aac.02071-21 PMC8923208

[8] Pirnay, J.-P. et al. Retrospective, observational analysis of the first one hundred consecutive cases of personalized bacteriophage therapy of difficult-to-treat infections facilitated by a belgian consortium. 10.1101/2023.08.28.23294728 (2023).

[9] Wdowiak, M., Paczesny, J. & Raza, S. Enhancing the stability of bacteriophages using physical, chemical, and nano-based approaches: A review. *Pharmaceutics* **14**, 1936 (2022). 36145682 10.3390/pharmaceutics14091936 PMC9502844

[10] Flint, R. et al. Stability considerations for bacteriophages in liquid formulations designed for nebulization. *Cells* **12**, 2057 (2023). 37626867 10.3390/cells12162057 PMC10453214

[11] Le Basle, Y., Chennell, P., Tokhadze, N., Astier, A. & Sautou, V. Physicochemical stability of monoclonal antibodies: A review. *J. Pharm. Sci.* **109**, 169–190 (2020). 31465737 10.1016/j.xphs.2019.08.009

[12] Raza, S., Wdowiak, M. & Paczesny, J. An overview of diverse strategies to inactivate enterobacteriaceae-targeting bacteriophages. *EcoSal Plus*10.1128/ecosalplus.esp-0019-2022 (2023). 36651738 10.1128/ecosalplus.esp-0019-2022 PMC10729933

[13] Chang, B. S. & Yeung, B. Physical stability of protein pharmaceuticals. In *Formulation and process development strategies for manufacturing biopharmaceuticals* (eds Jameel, F. & Hershenson, S.) 69–104 (Wiley, NJ, 2010). 10.1002/9780470595886.ch3.

[14] Akbarian, M. & Chen, S.-H. Instability challenges and stabilization strategies of pharmaceutical proteins. *Pharmaceutics* **14**, 2533 (2022). 36432723 10.3390/pharmaceutics14112533 PMC9699111

[15] Glonti, T. & Pirnay, J.-P. In vitro techniques and measurements of phage characteristics that are important for phage therapy success. *Viruses* **14**, 1490 (2022). 35891470 10.3390/v14071490 PMC9323186

[16] Khan Mirzaei, M. & Nilsson, A. S. Isolation of phages for phage therapy: A comparison of spot tests and efficiency of plating analyses for determination of host range and efficacy. *PLoS One* **10**, e0118557 (2015). 25761060 10.1371/journal.pone.0118557 PMC4356574

[17] Heider, S. & Metzner, C. Quantitative real-time single particle analysis of virions. *Virology* **462–463**, 199–206 (2014). 24999044 10.1016/j.virol.2014.06.005 PMC4139191

[18] Kaletta, J. et al. A rigorous assessment and comparison of enumeration methods for environmental viruses. *Sci. Rep.* **10**, 18625 (2020). 33122683 10.1038/s41598-020-75490-y PMC7596560

[19] Ács, N., Gambino, M. & Brøndsted, L. Bacteriophage enumeration and detection methods. *Front. Microbiol.* **11**, 68. 10.3389/fmicb.2020.594868 (2020). 33193274 10.3389/fmicb.2020.594868 PMC7644846

[20] Dharmaraj, Tejas et al. Rapid assessment of changes in phage bioactivity using dynamic light scattering. *PNAS Nexus*10.1093/pnasnexus/pgad406 (2023). 38111822 10.1093/pnasnexus/pgad406 PMC10726995

[21] Sausset, R. et al. Comparison of interferometric light microscopy with nanoparticle tracking analysis for the study of extracellular vesicles and bacteriophages. *J. Extracell. Biol.* **2**, e75 (2023). 38938523 10.1002/jex2.75 PMC11080698

[22] Turkki, V., Alppila, E., Ylä-Herttuala, S. & Lesch, H. P. Experimental evaluation of an interferometric light microscopy particle counter for titering and characterization of virus preparations. *Viruses* **13**, 939 (2021). 34069520 10.3390/v13050939 PMC8160961

[23] Cantero, M. et al. Atomic force microscopy of viruses: stability, disassembly, and genome release. In *Single molecule analysis: Methods and protocols* (eds Heller, I. et al.) 317–338 (Springer US, New York, NY, 2024). 10.1007/978-1-0716-3377-9_15. 10.1007/978-1-0716-3377-9_15 37824011

[24] Kuznetsov, Y. G., Chang, S.-C. & McPherson, A. Investigation of bacteriophage T4 by atomic force microscopy. *Bacteriophage* **1**, 165–173 (2011). 22164350 10.4161/bact.1.3.17650 PMC3225781

[25] Dubrovin, E. V. et al. Atomic force microscopy analysis of the acinetobacter baumannii bacteriophage AP22 lytic cycle. *PLOS ONE* **7**, e47348 (2012). 23071792 10.1371/journal.pone.0047348 PMC3469531

[26] Ackermann, H.-W. Bacteriophage electron microscopy. *Adv. Virus. Res.* **82**, 1–32 (2012). 22420849 10.1016/B978-0-12-394621-8.00017-0

[27] Brussaard, C. P. D. Enumeration of bacteriophages using flow cytometry. *Methods Mol. Biol.* **501**, 97–111 (2009). 19066815 10.1007/978-1-60327-164-6_11

[28] Roose-Amsaleg, C. et al. Utilization of interferometric light microscopy for the rapid analysis of virus abundance in a river. *Res. Microbiol.* **168**, 413–418 (2017). 28263904 10.1016/j.resmic.2017.02.004

[29] den Engelsman, J. et al. Strategies for the assessment of protein aggregates in pharmaceutical biotech product development. *Pharm. Res.* **28**, 920–933 (2011). 20972611 10.1007/s11095-010-0297-1 PMC3063870

[30] Pignataro, M. F., Herrera, M. G. & Dodero, V. I. Evaluation of peptide/protein self-assembly and aggregation by spectroscopic methods. *Molecules* **25**, 4854 (2020). 33096797 10.3390/molecules25204854 PMC7587993

[31] Raynal, B., Lenormand, P., Baron, B., Hoos, S. & England, P. Quality assessment and optimization of purified protein samples: Why and how?. *Microb Cell Fact* **13**, 180 (2014). 25547134 10.1186/s12934-014-0180-6 PMC4299812

[32] Gupta, A. & Mahalakshmi, R. Single-residue physicochemical characteristics kinetically partition membrane protein self-assembly and aggregation. *J. Biolog. Chem.* **295**, 1181–1194 (2020). 10.1074/jbc.RA119.011342 PMC6996891 31844019

[33] Abedon, S. T. & Katsaounis, T. I. Basic phage mathematics. In *Bacteriophages* (eds Clokie, Martha R.J.. et al.) 3–30 (Springer New York, New York, NY, 2018). 10.1007/978-1-4939-7343-9_1. 10.1007/978-1-4939-7343-9_1 29134583

[34] Cordova, A., Deserno, M., Gelbart, W. M. & Ben-Shaul, A. Osmotic shock and the strength of viral capsids. *Biophys. J.* **85**, 70–74 (2003). 12829465 10.1016/S0006-3495(03)74455-5 PMC1303066

[35] ICTV. Familiy Myoviridae. In ICTV (*International Committee on Taxonomy of Viruses*) Ninth Report; 2009 Taxonomy Release (2009).

[36] Szermer-Olearnik, B. et al. Aggregation/dispersion transitions of T4 phage triggered by environmental ion availability. *J. Nanobiotechnol.* **15**, 32 (2017). 10.1186/s12951-017-0266-5 PMC5404661 28438164

[37] Torisu, T., Maruno, T., Hamaji, Y., Ohkubo, T. & Uchiyama, S. Synergistic effect of cavitation and agitation on protein aggregation. *J. Pharm. Sci.* **106**, 521–529 (2017). 27887723 10.1016/j.xphs.2016.10.015

[38] Kolenda, C. et al. Phage therapy against staphylococcus aureus: Selection and optimization of production protocols of novel broad-spectrum Silviavirus Phages. *Pharmaceutics* **14**, 1885 (2022). 36145633 10.3390/pharmaceutics14091885 PMC9503876

[39] Austin, J., Minelli, C., Hamilton, D., Wywijas, M. & Jones, H. J. Nanoparticle number concentration measurements by multi-angle dynamic light scattering. *J. Nanopart. Res.* **22**, 108 (2020).

[40] Passaretti, P., Sun, Y., Dafforn, T. R. & Oppenheimer, P. G. Determination and characterisation of the surface charge properties of the bacteriophage M13 to assist bio-nanoengineering. *RSC Adv.* **10**, 25385–25392 (2020). 35517472 10.1039/d0ra04086j PMC9055230

[41] Kizuki, S., Wang, Z., Torisu, T., Yamauchi, S. & Uchiyama, S. Relationship between aggregation of therapeutic proteins and agitation parameters: Acceleration and frequency. *J. Pharm. Sci.* **112**, 492–505 (2023). 36167196 10.1016/j.xphs.2022.09.022

