# Supplementary material for: Real-time monitoring by interferometric light microscopy of phage suspensions for personalised phage therapy
Source: Sci Rep. 2024 Dec 30;14:31629. doi: 10.1038/s41598-024-79478-w (PMC11686143; doi:10.1038/s41598-024-79478-w)
Supplement: Supplementary file 1 — Supplementary Information 1. [file 41598_2024_79478_MOESM1_ESM.pdf]

Article

Real-time Monitoring by Interferometric Light Microscopy of Phage Suspensions for Personalised Phage Therapy

Benjamine Lapras [1,2], Camille Merienne [1], Emma Eynaud [1], Léa Usseglio [1], Chloé Marchand [1], Mathieu Médina [3], Camille Kolenda [3,4], Thomas Briot [5,6], Frédéric Laurent [3,4] & Fabrice Pirot [1,2] on behalf of PHAGE*in*LYON*

[1] Hospices Civils de Lyon, Hôpital E. Herriot, pharmacy department, Plateforme FRIPHARM, F-69437 Lyon, France; benjamine.lapras@chu-lyon.fr (B.L.); chloe.marchand@chu-lyon.fr (C.M.1); camille.merienne@chu-lyon.fr (C.M.2); emma.eynaud@chu-lyon.fr (E.E.); lea.usseglio@chu-lyon.fr (L.U.)

[2] Université Claude Bernard Lyon 1, Laboratoire de Recherche et Développement de Pharmacie Galénique Industrielle, Plateforme FRIPHARM, F-69008 Lyon, France ; CNRS UMR 5305, F-69007 Lyon, France; fabrice.pirot@univ-lyon1.fr (F.P.)

[3] Hospices Civils de Lyon, Hôpital Croix Rousse, bacteriology department, French National Reference Centre for Staphylococci, F-69317 Lyon, France; mathieu.media@chu-lyon.fr (M.M.); camille.kolenda@chu-lyon.fr (C.K.); frederic.laurent@univ-lyon1.fr (F.L.)

[4] Université Claude Bernard Lyon 1, International Centre for Research in Infectiology, F- 69365 Lyon, France; INSERM U1111, F- 69365 Lyon, France

[5] Hospices Civils de Lyon, Hôpital Croix Rousse, pharmacy department, F-69317 Lyon, France; thomas.briot@chu-lyon.fr (T.B.)

[6] Université Claude Bernard Lyon 1, Laboratoire d'Automatique, de Génie des Procédés et de Génie Pharmaceutique, CNRS UMR5007, 69622 Villeurbanne, France (T.B)

* A list of authors and their affiliations appears at the end of the paper

SUPPLEMENTARY INFORMATION

PHAGE*in*LYON:

Frédéric Laurent [3,4], Mathieu Medina [3], Camille Kolenda [3,4], Floriane Laumay [3,4], Mélanie Bonhomme [3], Leslie Blazere [3], Emilie Helluin [3], Charlotte Leravat [3], Alexia Von-Kanel [1], Fabrice Pirot [1,2], Camille Merienne [1], Benjamine Lapras [1,2], Chloé Marchand [1], Kévin Royet [4], Sylvain Goutelle [5], Romain Garreau [5],Gilles Leboucher [5], Thomas Briot [5,6], Ludivine Coignet [4], Myrtille Le Bouar [7], & Florent Valour [7]

[7] Department of Infectious Diseases, Hospices Civils de Lyon, F-69004 Lyon, France.

a

b

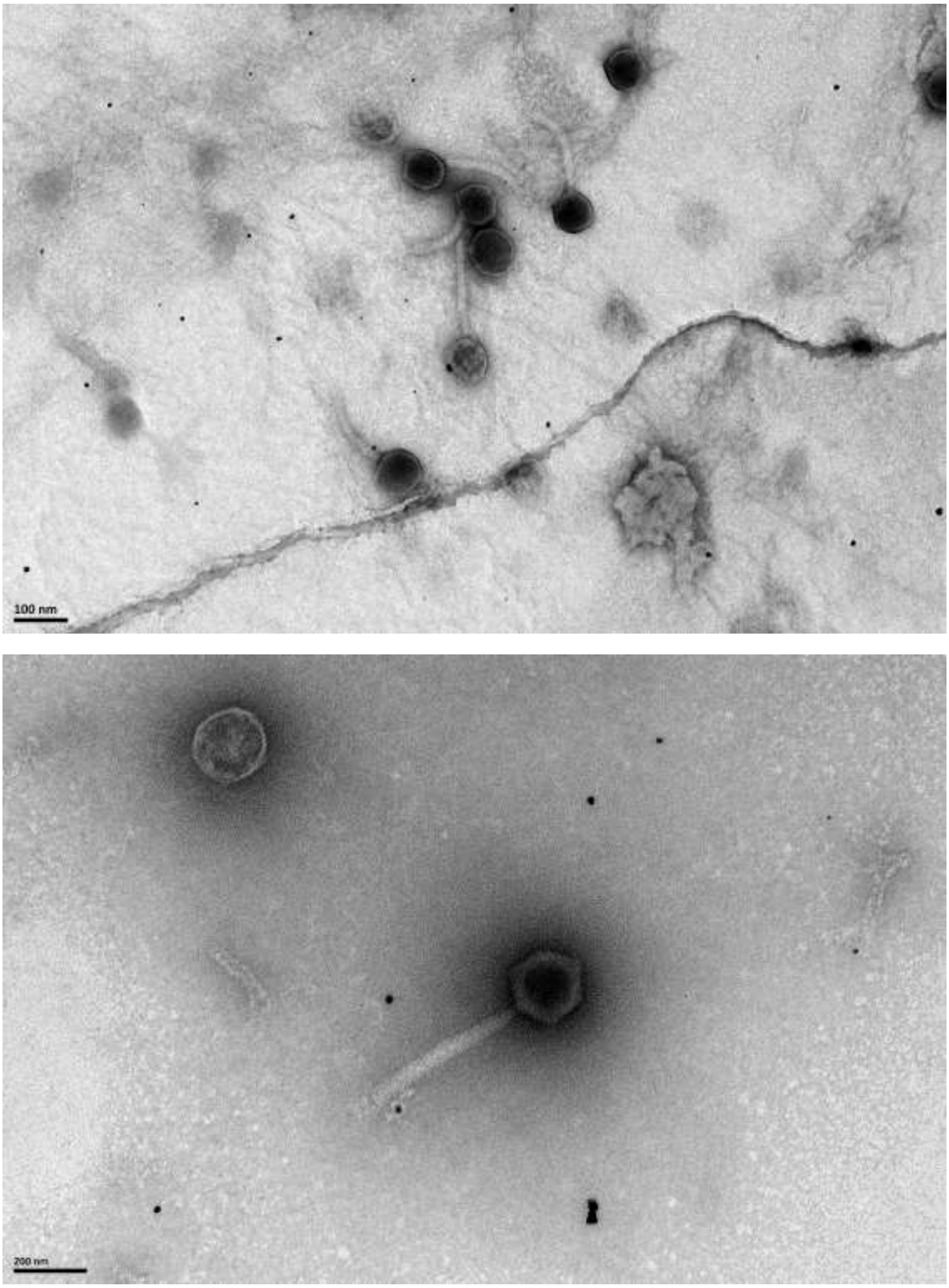

Fig. S1. TEM imaging of *Silviavirus* anti-*Staphylococcus aureus* phages **a** vB_SauM-V1SA19 (scale bar, 100 nm) and **b** vB_SauM-V1SA20 (scale bar, 200 nm), obtained as described in Kolenda et al., 2022.

Table 1. One way repeated measures ANOVAs comparing purification steps (PI, dPI, wPI, cPI, fPI) for each type of measure (phage titre, x; particle concentration, y).

	Degrees of freedom	*Sum of squares*	*Mean square*	*F*	*P-value*
Phage titre (x) *measures*					
Total	14	$2x10^{21}$	$1x10^{20}$		
Groups	4	$2x10^{21}$	$4x10^{20}$	71.12	<0.0001
Replicates	2	$2x10^{19}$	$8x10^{18}$	1.25	0.34
Error	8	$5x10^{19}$	$6x10^{18}$		
Particle concentration (y) *measures*					
Total	14	$1x10^{22}$	$9x10^{20}$		
Groups	4	$1x10^{22}$	$3x10^{21}$	35.75	<0.0001
Replicates	2	$2x10^{20}$	$1x10^{20}$	1.31	0.32
Error	8	$7x10^{20}$	$8x10^{19}$		

Homogeneity of variances was demonstrated beforehand with Levene's test (phage titre measures, p=0.29; particle concentration measures, p=0.26).

Table 2. In-process characterisation of purification replicate A by ILM and spot-test.

Particle characterisation	Purification steps				
	PI	dPI	wPI	cPI	fPI
ILM image	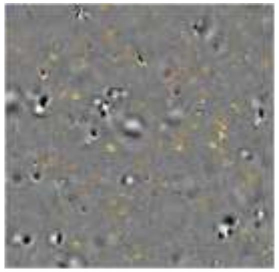	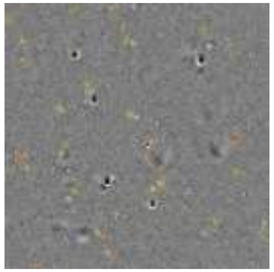	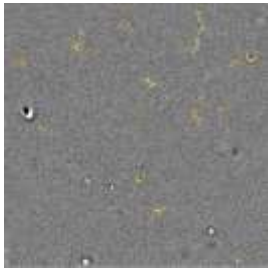	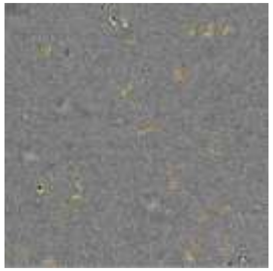	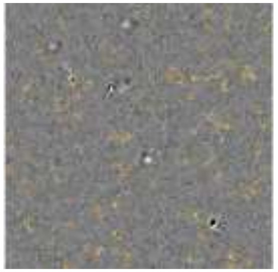
Infectious titre[‡] (PFU/mL)	(2.7 ± 0.7) 10^{10}	(2.2 ± 0.3) 10^{9}	(9.3 ± 5.0) 10^{8}	(1.9 ± 0.8) 10^{9}	(1.5 ± 0.8) 10^{10}
Particle concentration[†] (particles/mL)	(8.3 ± 0.1) 10^{10}	(5.4 ± 0.3) 10^{9}	(2.5 ± <0.1) 10^{9}	(3.4 ± <0.1) 10^{9}	(3.3 ± 0.2) 10^{10}
Mean particle diameter[†] (nm)	(168 ± 4)	(169 ± 8)	(169 ± 1)	(169 ± 1)	(155 ± 1)
Size distribution diagram	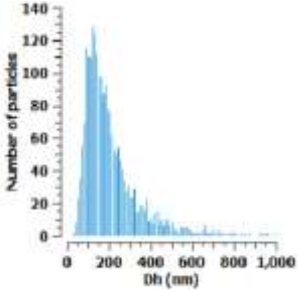	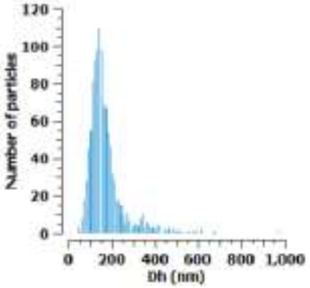	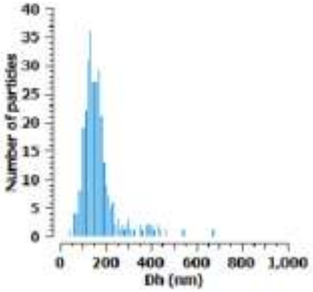	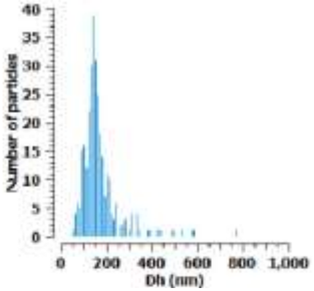	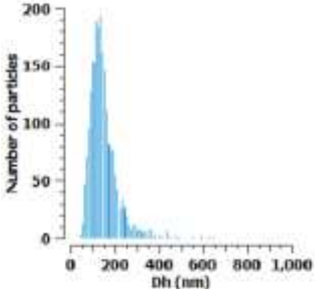

Each value is the (mean value ± sd) of 2 (†) to 3 (‡). ILM image and size distribution diagram were taken on undiluted samples while particle concentration and mean size were measured on samples diluted to fit the instrument specifications. The particle concentration displayed here is the detected value multiplied by the sample's dilution factor. On the ILM images, the orange lines circle tracked particles and the yellow lines track the particle movement.

Table 3. In-process characterisation of purification replicate C by ILM and spot-test.

Particle characterisation	Purification steps				
	PI	dPI	wPI	cPI	fPI
ILM image	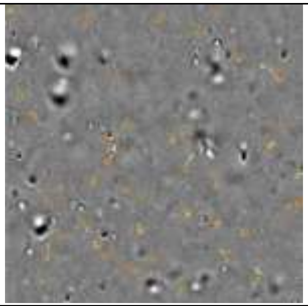	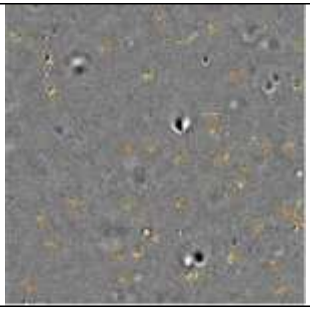	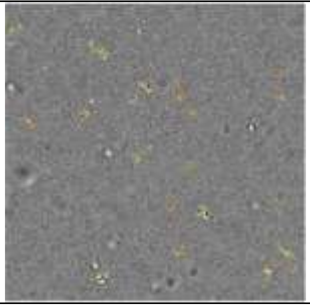	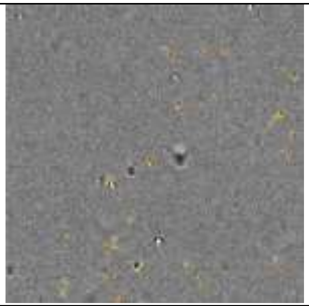	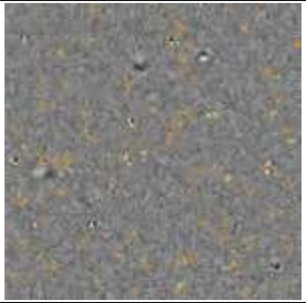
Infectious titre[ǂ] (PFU/mL)	(3.0 ± 1.0) 10^{10}	(2.7 ± 0.4) 10^{9}	(1.5 ± 0.8) 10^{9}	(6.0 ± 2.0) 10^{8}	(2.3 ± 0.4) 10^{10}
Particle concentration[†] (particles/mL)	(7.8 ± 0.8) 10^{10}	(1.0 ± <0.1) 10^{10}	(5.7 ± 0.2) 10^{9}	(4.9 ± 1.6) 10^{9}	(6.5 ± 1.1) 10^{10}
Mean particle diameter[†] (nm)	(184 ± 1)	(174 ± 6)	(174 ± 11)	(157 ± 5)	(155 ± 4)
Size distribution diagram	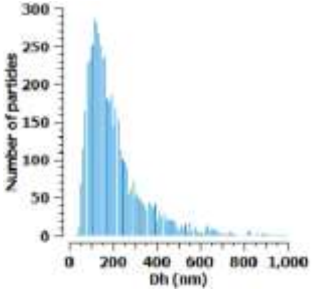	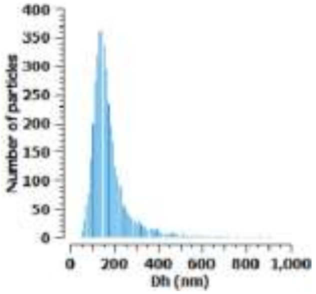	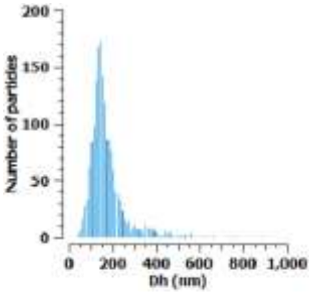	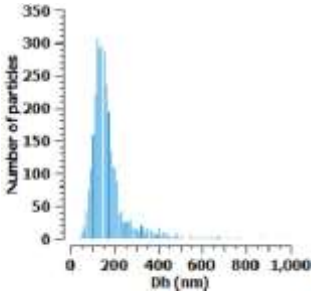	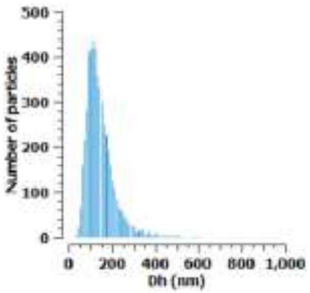

Each value is the (mean value ± sd) of 2 (†) to 3 (ǂ). ILM image and size distribution diagram were taken on undiluted samples while particle concentration and mean size were measured on samples diluted to fit the instrument specifications. The particle concentration displayed here is the detected value multiplied by the sample's dilution factor. On the ILM images, the orange lines circle tracked particles and the yellow lines track the particle movement.
